# Gastropleural Fistula Presenting as a Complication of Gastric Sleeve Surgery: A Case Report

**DOI:** 10.7759/cureus.37133

**Published:** 2023-04-04

**Authors:** Bilal Koussayer, Mafaz Kattih, Matthew Nester, Pete Peterson, Christopher G DuCoin

**Affiliations:** 1 Surgery, University of South Florida Morsani College of Medicine, Tampa, USA; 2 General Surgery, Tampa General Hospital, Tampa, USA

**Keywords:** robotic surgical procedures, post operative complication, gastric sleeve, gi fistulas, gastropleural fistula

## Abstract

A rare complication of sleeve gastrectomy surgery is gastropleural fistulas (GPF), where a fistula develops between the stomach and the pleural cavity. This complication can be debilitating and present with many nonspecific symptoms making it hard to diagnose. This is a case report of a 45-year-old female who underwent robotic-assisted gastric sleeve revision after developing a GPF as a complication of her gastric sleeve six years later. This led to the development of a recurrent subdiaphragmatic abscess in the left upper quadrant. Before presenting to us, she underwent multiple hospitalizations and received numerous endoscopic stent treatments. However, the abscess continued to recur. Given her recurrent abscess, she consented to gastric sleeve revision. GPFs are amongst the rarest complications, with only 76 reported cases. Since this complication can cause shock, early diagnosis and treatment are necessary to improve patient outcomes and reduce morbidity.

## Introduction

Sleeve gastrectomy is a popular weight loss surgery that has grown over the last 10 years, emerging as one of the standard treatment options for obese patients. The surgery involves minimally invasive laparoscopic surgery in which the part of the stomach that is responsible for appetite regulation is resected [1]. Compared to Roux-en-Y Gastric Bypass, it is much less technically challenging as no anastomosis or bypass needs to be made [1]. Although now common practice, it is not without its complications, of which pulmonary complications are usually rare. Amongst the rarest complications are gastropleural fistulas (GPFs), where communication develops between the stomach and the pleural space. This complication can cause debilitating pain in patients and present with nonspecific symptoms such as chest pain, shortness of breath, and cough. Diagnosis is best made using a CT scan with contrast. Other GI imaging modalities, like MRI, can aid with evaluating GI fistulas and can be used for better diagnostic practices in detecting GPF. Since this complication can cause septic shock, early diagnosis and effective treatment are necessary to improve patient outcomes and reduce morbidity. A systematic review published in 2021 reveals that this complication has only been reported in 76 patients [2]. Based on the rarity of the complication, this case report details the presentation and outcome of a 45-year-old female diagnosed with a GPF.

## Case presentation

**Figure 1:**
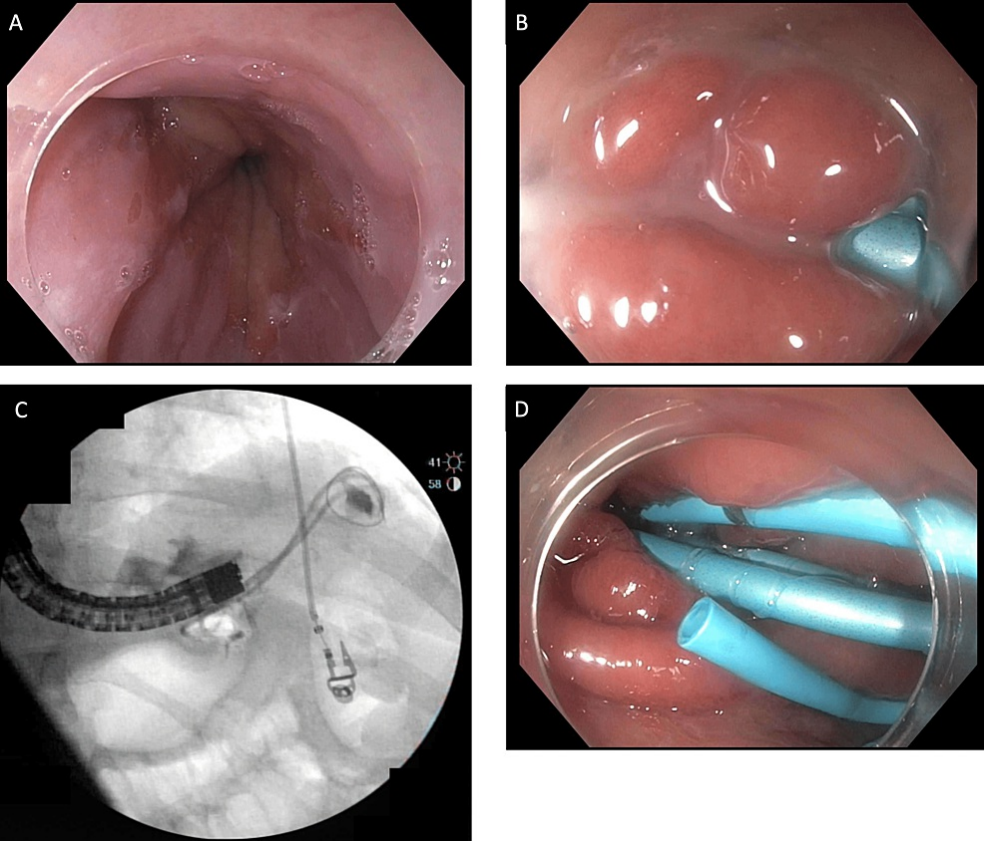
Upper gastrointestinal endoscopy. Preoperative upper GI endoscopy. A) Body of the stomach. B) Small amount of puss draining. C) Wire placement followed by contrast injection. D) Three 7 Fr stents were placed transmurally.

**Figure 2:**
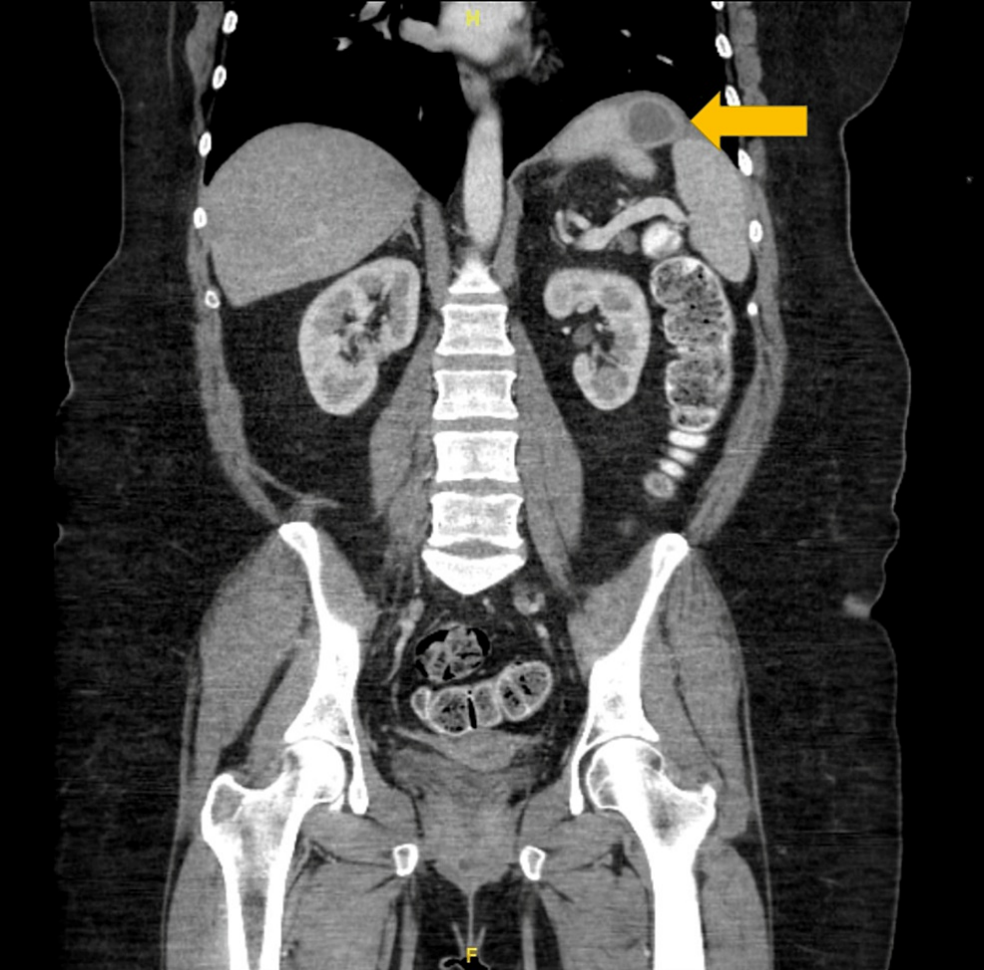
Preoperative CT scan. Coronal CT scan showing sub-diaphragmatic abscess (arrow) secondary to gastropleural fistula.

A 45-year-old female with a past medical history of coronary artery disease and hypothyroidism presented with back and left flank pain, phrenic nerve irritation leading to left shoulder pain, and pain during breathing. Past surgical history included a gastric band that was placed in 2007, which was converted to a gastric sleeve in 2012. The patient then developed a subdiaphragmatic abscess in the left upper quadrant and underwent several hospitalizations and multiple endoscopic stent treatments (Figures 1A-1D). However, the abscess continued to reaccumulate. A CT was done, revealing the abscess, as seen in Figure 2. The patient consented to robotic-assisted laparoscopic revision of her gastric sleeve and drainage of abdominal abscess with drain placement.

The patient was brought to the operating room (OR), and the abdomen was entered and insufflated with a 5 mm Optiview Trocar to 15 mm Hg. An esophagogastroduodenoscopy (EGD) was performed, identifying a draining fistula 2 cm distal to the gastroesophageal (GE) junction with puss in the gastric sleeve (Video 1). A redundant fundus was also present at the level of the fistula. Through CT-guided imaging, the abscess cavity was found and drained (Video 1).

**Video 1:** Robotic-assisted gastropleural fistula repair. Intraoperative video of how gastropleural fistula was treated using robotic surgery.

he abscess was then fenestrated and resected. The resection area and pleural abscess were connected to make one large cavity denuded of necrotic tissue. The fundus of the stomach was mobilized, and the diseased fistula was resected using a 60 mm endo GIA blue load stapler that was then sewn over with 2-0 silk. A leak test was performed, and no leaks were noted, confirming that the fistula had been resected. The patient was extubated in the OR and sent to the post-anesthesia care unit (PACU), alert, awake, and spontaneously respirating. POD#1 fluoroscopic upper GI series showed that the surgery was successful, and no leakage was visualized. The patient’s post-operative course was unremarkable. The patient was seen in the clinic for follow-up, and her symptoms were resolved.

## Discussion

GPFs occur when there is communication between the stomach and the pleural space. This pathological condition was first described in 1960 by Markowttz and Herter, but a 2021 systematic review revealed that there had been 76 patients with post-bariatric gastric fistulas in the literature, making this complication a rare and little-discussed one [2]. The etiology of GPFs is elusive, yet bariatric surgery and sleeve gastrectomy have been cited as causes of the complications. Detecting GPFs is difficult as the patients can present with nonspecific symptoms such as productive cough, shortness of breath, chest pain, or abdominal pain. In this case, the patient also had referred pain in her left shoulder and flank. Due to the variable symptoms, imaging is essential in detecting GPFs, with CT imaging and MRIs being at the forefront of diagnosis [3]. This case utilized MRI and CT to identify the abscess and its location before surgery. There is a scarcity of guidelines on how GPFs should be managed. Some argue for a laparoscopic approach, while others a more conservative approach with antibiotic use and percutaneous drainage [2]. Shoar S et al. describe a step-up approach for treatment, first with noninvasive measures, then minimally invasive procedures, and finally surgical intervention, which yields better visualization and better instrument control. Furthermore, Shoar S et al. cite a study where 20% of patients saw improvement with conservative treatment over the course of three months [4]. After those three months, conservative treatment could be considered as having a limited effect, and it was best to move to surgical intervention if there was no improvement [4]. As this patient had a long history of abscess accumulation despite both conservative management and surgical procedures, it was clear that the more conservative treatment was not working. Thus, a surgical revision of the gastric sleeve was needed to treat the issue. One of the first cases of robotic laparoscopy for GPFs is described in the study by Garcia-Quintero P et al., and the laparoscopic approach was shown as safe and feasible after the failure of conservative management and traditional treatment methods. Robotic laparoscopy was chosen as the preferred method due to its benefits in visualization and tremor control with few impactful limitations, including space required and lack of haptic feedback [5]. However, the impact of these limitations can be readily decreased, leaving robotic laparoscopy as a safe approach for GPFs and other bariatric surgeries.

## Conclusions

In this case, the patient underwent a gastric sleeve revision, and the GPF was treated using robotic laparoscopy. Previous attempts at treating the patient’s abscess were unsuccessful, as the abscess was recurring despite minimally invasive procedures. The patient underwent three different EGD with stent insertions in 2021; however, they did not resolve the issue and thus were of limited use. This led to the necessity of surgical intervention, which corresponds to the progressive treatment approach Shoar S et al. described. In conclusion, successful management of GPF includes not only early detection but early surgical interventions to reduce morbidity and mortality.
